# Association Between Early Vascular Aging and Cardiometabolic Diseases: A Two-Year Longitudinal Study of the EVasCu Cohort

**DOI:** 10.3390/jcm15145520

**Published:** 2026-07-14

**Authors:** Marta Fenoll-Morante, Alicia Saz-Lara, Arturo Martinez-Rodrigo, Nerea Moreno-Herraiz, Iris Otero-Luis, José Alberto Martínez-Hortelano, Carla Geovanna Lever-Megina, Iván Cavero-Redondo

**Affiliations:** 1 CarVasCare Research Group, Instituto de Biomedicina, Facultad de Enfermería de Cuenca, Universidad de Castilla-La Mancha, 16071 Cuenca, Spain; martafenoll6@gmail.com (M.F.-M.); nerea.moreno@uclm.es (N.M.-H.); iris.otero@uclm.es (I.O.-L.); ivan.cavero@uclm.es (I.C.-R.); 2 Research Group in Medical Informatics, E-Health and Advances Technologies, Universidad de Castilla-La Mancha, 16071 Cuenca, Spain; arturo.martinez@uclm.es; 3 Faculty of Nursing, Universidad de Castilla-La Mancha, 02006 Albacete, Spain

**Keywords:** advanced glycation and products, aortic pulse wave velocity, cardiovascular disease, early vascular aging, glycated hemoglobin and pulse pressure

## Abstract

**Background.** Cardiometabolic diseases are the leading cause of morbidity and mortality worldwide and are driven by risk factors such as hypertension, diabetes mellitus, and dyslipidemia. Early vascular aging (EVA) is a pathological process characterized by accelerated arterial stiffness and endothelial dysfunction, which increase the risk of cardiovascular events. Key markers of EVA include advanced glycation end products (AGEs), aortic pulse wave velocity (a-PWV), glycated hemoglobin A1c (HbA1c) and pulse pressure (PP). Although these markers are individually associated with cardiovascular outcomes, there is still limited evidence from longitudinal studies evaluating their combined association with cardiometabolic diseases and hypertension, particularly with consideration of gender differences. Therefore, in this study, data from the two-year EVasCu cohort were used to analyze the associations between EVA and cardiometabolic diseases and hypertension; the associations between EVA related parameters (AGEs, a-PWV, HbA1c and PP) were evaluated, and these associations were assessed by gender. **Methods.** AGE, a-PWV, HbA1c and PP were measured as indicators of EVA, in addition to sociodemographic and clinical variables. Logistic regression was applied to assess the association between EVA and cardiometabolic diseases (including hypertension, diabetes mellitus, acute myocardial infarction (AMI), dyslipidemia, angina pectoris, and heart failure (HF)) and hypertension, adjusting for age, gender and risk factors. **Results.** A 2-year longitudinal study was conducted with 200 adults in Cuenca, Spain. EVA was significantly associated with the development of cardiometabolic diseases (OR = 1.38; *p* = 0.028) and hypertension (OR = 1.63; *p* = 0.015), which was more pronounced in females. Cardiometabolic diseases and hypertension were associated with a-PWV and PP, but not with AGEs or HbA1c. **Conclusions.** a-PWV and PP are predictors of cardiometabolic diseases and hypertension, especially in females. Early detection and preventive strategies are essential for detecting cardiovascular risk and mitigating consequences. Further studies are needed to investigate the relationships between EVA and cardiometabolic factors.

## 1. Introduction

Cardiometabolic diseases, which include cardiovascular disease (CVD), diabetes mellitus, hypertension and metabolic syndrome, are the leading causes of mortality and morbidity worldwide, contributing to a significant increase in healthcare costs [1]. The number of deaths attributed to cardiometabolic diseases increased from 12.1 million in 1990 to 18.6 million in 2019 [2]. Several studies have indicated that cardiovascular risk factors such as hypertension, diabetes mellitus and dyslipidemia are responsible for premature structural and functional changes at the vascular level, known as early vascular aging (EVA) [3,4]. Healthy lifestyle habits can contribute to a reduction in the risk of multimorbidity of cardiometabolic diseases, even before the manifestation of some conditions, such as these habits [5].

EVA is related mainly to increased arterial stiffness, which is a predictor of cardiovascular morbidity and mortality, as well as an early indicator of atherosclerosis. EVA is a construct consisting of health markers associated with cardiovascular morbidity and mortality, and it has recently been established that EVA consists of aortic pulse wave velocity (a-PWV), pulse pressure (PP), glycated hemoglobin A1c (HbA1c) and advanced glycation end products (AGEs) [6,7,8]. In this context, EVA has been described even in apparently healthy individuals without traditional cardiovascular risk factors, reinforcing the idea that arterial stiffness and vascular dysfunction often precede overt clinical disease [9,10]. Carotid-Femoral PWV is the most accessible, reproducible, noninvasive marker for assessing arterial stiffness and is considered the gold standard for its evaluation [11], as well as an independent predictor of cardiovascular events [10]. Several studies have shown that both a-PWV and PP are independent predictors of cardiovascular events, encompassing a wide clinical spectrum that ranges from healthy individuals to high-risk individuals [10,11]. Additionally, it has been suggested that hyperglycemia induces important quantitative and qualitative changes in elastin and collagen in central arterial walls. Finally, AGEs form cross-links in collagen fibers, decreasing the distensibility of arterial walls [12,13]. Furthermore, HbA1c is a key and essential marker for assessing glycemic control and cardiovascular risk in individuals with diabetes mellitus and other metabolic disorders [14,15,16].

Furthermore, high BP is among the most prevalent risk factors and contributes significantly to the development of arterial stiffness, thereby increasing the likelihood of CVD [10,17]. Studies have also shown that physiological and clinical risk factors are related to both genetic and environmental factors and that prolonged accumulation of these factors and lifestyle habits are determinants of the development of long-term cardiovascular events, with early onset at an early age [18,19]. However, it is recognized that there is a discrepancy between chronological age and vascular age, since in some individuals, vascular aging occurs at an accelerated rate, increasing the risk of developing CVD [20]. This relationship is increasingly understood to be bidirectional, as arterial stiffness is not only a consequence of elevated blood pressure but also an independent determinant of hypertension development [21]. Although current evidence suggests that there may be a direct relationship between cardiometabolic diseases, hypertension, and early vascular aging (EVA) [22], studies specifically analyzing the association between EVA-related parameters (a-PWV, AGEs, HbA1c and PP) and cardiometabolic diseases and hypertension are lacking. Further research, particularly longitudinal studies, is needed to better understand this relationship and its potential clinical implications.

Therefore, this study, using data collected from a two-year longitudinal study of the EVasCu cohort, aimed to: (i) analyze the association between EVA and cardiometabolic diseases and hypertension; (ii) analyze the association between EVA-related parameters (a-PWV, AGEs, HbA1c and PP) and cardiometabolic diseases and hypertension; and (iii) analyze the association of these variables with both cardiometabolic diseases and hypertension by gender.

## 2. Methods

### 2.1. Design, Participants and Sample Size

The EVasCu study is a two-year longitudinal study that collected data from healthy adults in the city of Cuenca, Spain. These participants were initially recruited on 1 June 2022, and the final measurement was taken on 1 June 2024. The follow-up period lasted two years.

The inclusion criteria were as follows: (1) adult subjects (aged over 18 years) who were apparently healthy (without diagnostic criteria for chronic disease). (2) who were clinically stable 6 weeks prior to the start of the study, and (3) who provided written informed consent. The exclusion criteria for participants included: (1) participation in another study, (2) the presence of diagnosed pathologies at the start of the study and (3) being under pharmacological treatment at the start of the study. This study was conducted in accordance with the guidelines for reporting observational studies Strengthening the Reporting of Observational Studies in Epidemiology (STROBE) [23].

The sample size was calculated using Epidat software, version 4.2., which indicated that 355 participants would provide an estimated effect size of 1, with a risk alpha of 0.05 and an absolute precision level of 0.04 to detect a statistically significant result for the EVA index [24]. Participants were invited to enroll in the study on the basis of the inclusion and exclusion criteria, and 390 participants were ultimately enrolled.

**Figure 1:**
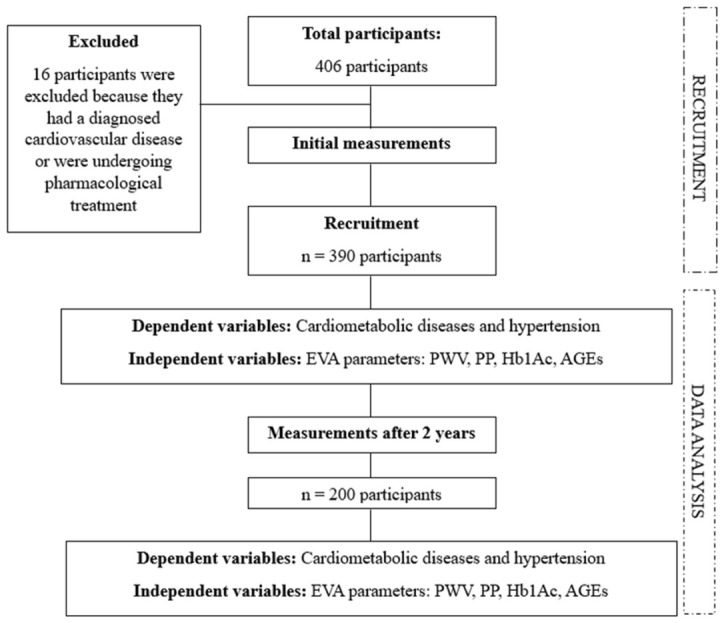
Flow diagram. AGEs: Advanced glycation end-products; EVA: Early vascular aging; HbA1c: Glycated hemoglobin; PP; Pulse pressure; PWV; Pulse wave velocity.

The following figure shows the flow chart with the criteria used for inclusion/exclusion, recruitment and measurement criteria for the subjects included in the study. The study began with 406 participants, 16 of whom were excluded because they had been diagnosed with chronic diseases at baseline. This resulted in a sample of 390 apparently healthy participants at the start of the study. Two years later, the same measurements were taken to determine whether the participants assessed at the start of the study had developed cardiometabolic diseases and/or hypertension (Figure 1).

### 2.2. Ethical Considerations

The research protocol for this study was approved by the Cuenca Area Health Clinical Research Ethics Committee (REG: 2023/PI1823). Written informed consent to participate was obtained from all the subjects included in the study. All procedures performed in this study were in accordance with the Declaration of Helsinki and its subsequent amendments or comparable ethical standards for experiments involving humans [25].

The study initially received approval from the Ethics Committee for Research (CEI) of the Cuenca Health Area (approval code: REG: 2022/PI2022) on 18 May 2022. Participant recruitment began on 1 June 2022 under this approval. Subsequently, a follow-up assessment of the previously recruited participants was conducted. A new ethical approval was obtained from the Ethics Committee for Research with Medicines (CEIm) of the Cuenca Health Area (approval code: REG: 2023/PI1823) on 28 August 2023.

### 2.3. Study Variables

#### 2.3.1. Independent Variables

The EVA construct was operationalized using four previously described EVA-related parameters (a-PWV, AGEs, HbA1c and PP) [6,7,8]. All four variables were entered into the model in their original measurement units and were subsequently standardized using z-scores to avoid scale-dependent weighting. In this respect, EVA/HVA status was derived using an unsupervised K-means clustering model with two clusters based on these four parameters. Participants were assigned to the nearest cluster centroid according to the Euclidean distance in the standardized four-dimensional space. The cluster characterized by higher standardized values of a-PWV, AGEs, HbA1c, and PP was labeled as EVA, whereas the cluster with lower standardized values was labelled as healthy vascular aging (HVA).

It is worth noting that no predefined clinical cut-off points were used for the individual parameters. Instead, EVA/HVA status was determined by the multivariate clustering structure. For transparency and interpretability, the equivalent decision rule in the original measurement units was calculated from the standardized centroids and scaler parameters. Thus, participants were classified as EVA when:

1.014255 × a-PWV + 3.221062 × AGEs + 3.426339 × HbA1c + 0.045758 × PP > 32.7863

Participants not satisfying this rule were classified as HVA.

A-PWV was measured by oscillometric techniques using Mobil-O-Graph equipment (IEM GmbH, Stolberg, Germany), which assesses the PWV at the aortic level (a-PWV). This parameter was calculated as the mean of two repeated measurements, separated by 5 min each. It was measured in a quiet place, using a cuff size appropriate to the circumference of the participant’s arm.

PP was obtained from the difference between systolic blood pressure (SBP) and diastolic blood pressure (DBP). Two BP measurements were taken at 3 min intervals, in a quiet place and after a 5 min rest period using an Omron^®^ M5-I monitor (Omron Healthcare UK Ltd., Buckinghamshire, UK) with a cuff size according to the circumference of the participant’s upper arm.

HbA1c was determined by high-performance liquid chromatography using the ADAMS A1c analyzer HA-8180 V from A. Menarini Diagnostics^®^, Florence, Italy. Blood samples were obtained from a vein in the antecubital fossa, between 8 and 9 am and after 12 h of fasting.

AGEs were measured by skin autofluorescence (SAF) with an AGE Reader^®^ device, AGEs, Groningen, The Netherlands. AGEs were calculated as the means of the measurements from both arms. The mean of each arm was calculated as the mean of three repeated measurements.

#### 2.3.2. Dependent Variables

− *Hypertension*

Hypertension status was established according to the SBP and DBP criteria of the American Heart Association (AHA) and the American College of Cardiology (ACC) [26]. In accordance with these criteria, the following was established: optimal BP (SBP < 120 mmHg and/or DBP < 80 mmHg), normal BP (SBP 120–129 mmHg and/or DBP 80–84 mmHg), normal-high BP (SBP 130–139 mmHg and/or DBP 85–89 mmHg), grade I HBP (SBP 140–159 mmHg and/or DBP 90–99 mmHg), grade II HBP (SBP ≥ 160 mmHg and/or DBP ≥ 100 mmHg) and grade III HBP (SBP ≥ 180 mmHg and/or DBP ≥ 110 mmHg).

− *Cardiometabolic diseases*

The cardiometabolic disease variable was obtained in a self-reported manner after 2 years of follow-up and included the following pathologies: hypertension, diabetes mellitus, acute myocardial infarction (AMI), dyslipidemia, angina pectoris and heart failure (HF).

The diseases included were identified on the basis of the information provided by the participants themselves in their medical histories.

#### 2.3.3. Covariables

− *Sociodemographic factors and lifestyles*

Age, gender, smoking status, educational level and family history were collected by a self-report questionnaire. For gender, the possible responses were male or female.

Smoking status was classified into five groups: smokers, ex-smokers < 1 year, ex-smokers aged 1–5 years, ex-smokers > 5 years and nonsmokers.

Educational level was measured by six groups: cannot read or write, no education, primary education, secondary education, vocational education and university degree. For this analysis, the categories not able to read or write, no education, secondary education and vocational training were merged, resulting in four groups.

With respect to family history, participants were asked whether they had a history of stroke or AMI. The age at the time of the event and the gender of the person were specified.

− *Adiposity factors*

Body fat percentage was measured by calculating the average of two measurements using a Tanita BC-418 MA 8-electrode electrical bioimpedance.

### 2.4. Statistical Analysis

Normal probability plots and the Shapiro–Wilk test were used to verify the normality of the distribution of continuous variables. Descriptive statistics for the first measurement database are presented as the means and standard deviations (SDs) or proportions (%), as appropriate, for the total sample. For continuous variables, Student’s *t*-test for independent samples was used, and variables were grouped by gender. For categorical variables, the chi2 test was used, as was the use of gender.

A binary logistic regression model was used to explore how the dichotomous categorical dependent variables (cardiometabolic diseases and hypertension) were related to the independent variables (EVA construct, a-PWV, AGEs, HbA1c and PP). This analysis was performed using four adjustment models: (1) unadjusted model; (2) adjusted model 1, by age and gender; (3) adjusted model 2, by age, gender, fat percentage and educational level; and (4) adjusted model 3, by age, gender, fat percentage, educational level, family history and current smoking status. All the above analyses were also performed by gender. In all analyses, when segmented by gender, male and female, in models 1, 2 and 3, the gender variable was removed from the adjustment.

Prior to multivariable logistic regression modeling, multicollinearity among the independent variables included in the adjusted models was assessed using variance inflation factors (VIFs) and tolerance statistics. Covariates were selected a priori on the basis of their established associations with vascular aging and cardiometabolic outcomes, rather than through data-driven selection procedures. A VIF > 5 was considered indicative of potentially problematic multicollinearity.

Statistical analyses were performed using IBM SPSS version 28, and *p* < 0.05 was considered to indicate statistical significance.

## 3. Results

### 3.1. Characteristics of the Included Population

The initial sample in the EVasCu study included a total of 390 participants, of whom 200 subjects remained after 2 years of follow-up (Figure 1), at which point measurements were taken from all of them.

Among the initial sample, 246 (63.1%) were female and the mean age of the participants was 42.02 ± 13.14 years. In terms of body fat, a body fat percentage of 27.25 ± 9.36 was observed in the total population, with a significantly greater body fat percentage (*p* < 0.001) in females (31.54 ± 7.82) that in males (19.92 ± 6.91).

In addition, 17.2% of the participants reported that they had a family history of stroke, and statistically significant differences (*p* = 0.010) were observed between women (21%) and men (10.6%) On the other hand, 26.7% reported a family history of AMI. With respect to smoking habits, 12.6% of the total population were current smokers. A total of 57.3% of the participants had a university degree, while 21.8% had completed primary, secondary, vocational or high school.

In terms of cardiovascular parameters, the a-PWV was significantly different (*p* = 0.037) between males (6.53 ± 1.34) and female (6.23 ± 1.35). Moreover, PP was significantly greater (*p* < 0.001) in males (52.45 ± 9.76) than in females (42.50 ± 8.50). On the other hand, for HbA1c and AGEs no statistically significant differences were observed. HR values were significantly greater (*p* < 0.001) for women (69.31 ± 10.60) than for men (63.35 ± 10.17). In contrast, the SBP was significantly greater (*p* < 0.001) in men (125.09 ± 12.76) than in women (111.77 ± 14.37). The same pattern was observed for DBP, which was also significantly greater (*p* = 0.006) in men (72.27 ± 10.32) that in women (69.24 ± 10.58 mmHg). Using this cluster-based definition, 164 participants (42.1%) were classified as EVA at baseline, whereas 226 participants (57.9%) were classified as HVA. Compared with participants with HVA, those with EVA had significantly higher a-PWV, AGEs, PP, HbA1c, SBP, and DBP, both at baseline and after the two-year follow-up.

At the 2-year follow-up, significant sex differences in family history of stroke were observed (*p* = 0.041), with a higher prevalence among women (26.2%) than among men (13.5%). In addition, the HR remained significantly greater (*p* = 0.001) for women (71.42 ± 11.72) than for men (65.88 ± 10.82). In contrast, the SBP was significantly greater (*p* < 0.001) in men (127.07 ± 12.50) that in women (113.64 ± 12.00). Similarly, DBP was significantly greater (*p* = 0.014) in men (73.84 ± 8.71) than in women (70.38 ± 8.81). a-PWV was also significantly greater (*p* = 0.003) in men (7.16 ± 1.49) that in women (6.51 ± 1.38). Similarly, the PP was significantly greater (*p* = 0.011) in men (53.22 ± 10.66) than in women (43.26 ± 7.60 mmHg).

At the 2-year follow-up, 25 participants developed cardiometabolic diseases, of whom 10 were men and 15 were women. In addition, 7 participants (4 men and 3 women) developed hypertension (Table 1).

### 3.2. Associations Between EVA and Cardiometabolic Diseases and Hypertension

The associations between EVA and cardiometabolic diseases and hypertension are shown in Table 2. The EVA showed an OR of 1.38 for cardiometabolic diseases (*p* = 0.028) and an OR of 1.63 for hypertension (*p* = 0.015) in the unadjusted model in the total population. This association was maintained in females in the unadjusted model, with an OR of 1.91 for cardiometabolic diseases (*p* = 0.010) and an OR of 4.07 for hypertension (*p* = 0.003). For Model 1, adjusted for age in females, an OR of 3.77 for hypertension (*p* = 0.030) was observed.

### 3.3. Associations Between a-PWV and Cardiometabolic Diseases and Hypertension

The associations between a-PWV and cardiometabolic diseases and hypertension are shown in Table 3. a-PWV was associated with hypertension in the unadjusted model (OR = 2.08; *p* = 0.002), model 1 (OR = 5.56; *p* = 0.002), model 2 (OR = 5.67; *p* = 0.003) and model 3 (OR = 19.6; *p* < 0.001). In addition, a-PWV had an OR of 1.53 for cardiometabolic diseases (*p* = 0.004) in the unadjusted model for the total population. In females, a-PWV was associated with hypertension in the unadjusted model (OR = 4.45; *p* = 0.010), model 1 (OR = 11.81; *p* = 0.041) and model 3 (OR = 14.41; *p* = 0.031). Additionally, a-PWV had an OR of 1.82 for cardiometabolic diseases (*p* = 0.006) in the unadjusted model for females.

### 3.4. Associations Between PP and Cardiometabolic Diseases and Hypertension

The associations between PP and cardiometabolic diseases and hypertension are shown in Table S1. PP was associated with hypertension in the unadjusted model (OR = 1.07; *p* = 0.009), model 1 (OR = 1.06; *p* = 0.046) and model 3 (OR = 1.09; *p* = 0.019) in the total population. On the other hand, PP had an OR of 1.14 for hypertension (*p* = 0.008) in the unadjusted model for females.

### 3.5. Associations Between AGEs and Cardiometabolic Diseases and Hypertension

Table S2 shows the associations between AGEs cardiometabolic diseases and hypertension. AGEs were not significantly associated with cardiometabolic diseases and hypertension.

### 3.6. Associations Between HbA1c and Cardiometabolic Diseases and Hypertension

The associations between HbA1c and cardiometabolic diseases and hypertension are shown in Table S3. HbA1c was not significantly associated with cardiometabolic diseases and hypertension.

In all analyses, when segmented by gender, men and women, in models 1, 2 and 3 the gender variable was removed from the adjustment.

For all regression models, no evidence of substantial multicollinearity was observed among the covariates included in the adjusted models. The VIF ranged from 1.07 to 1.72, and all the tolerance values exceeded 0.20, indicating acceptable levels of collinearity.

Table 4 shows the absolute changes in the main variables of the EVA construct after a two-year follow-up compared to baseline values, both for the overall population and for the population stratified by gender. At the two-year follow-up, a-PWV showed a significant increase in both men and women, with a greater change observed in men (Δ = 0.24, *p* < 0.001) compared with women (Δ = 0.15, *p* < 0.001). AGEs showed a small but significant decrease only in men (Δ = −0.12, *p* < 0.001), while no significant changes were observed in the overall population or in women. Finally, no significant changes were observed for PP or HbA1c in either the general population or by gender.

Table 5 compares cardiovascular parameters between individuals with EVA and those with HVA, both at baseline and after 2 years of follow-up. Compared with the HVA group, the EVA group had significantly higher a-PWV, AGEs, PP, HbA1c, SBP and DBP (all *p* < 0.001). HR did not significantly differ between groups at either baseline or follow-up. Finally, an analysis of the absolute changes over the two-year period revealed no significant differences in the progression of any of the parameters evaluated between the two groups.

## 4. Discussion

The objectives of this study were to analyze the associations between EVA and EVA-parameters (a-PWV, AGEs, HbA1c and PP) and cardiometabolic diseases and hypertension and to analyze these associations by gender. After the results of the EVasCu study were analyzed, it was observed that EVA predicted 38% of the risk of cardiometabolic diseases and 63% of the risk of hypertension at 2-years of follow-up, but these associations were greater for females. In addition, females with EVA were 2 times more likely to develop cardiometabolic disease and 4 times more likely to develop hypertension. On the other hand, a-PWV was associated with cardiometabolic diseases and hypertension, and in addition, PP was associated with hypertension, with the association being greater in females. It is important to note that the EVA/HVA classification was derived from a model that grouped participants based on the overall pattern of the four parameters related to EVA (VOP, AGE, HbA1c, and PP) and, based on the results obtained, serves as a strong predictor of cardiometabolic diseases.

In relation to this analysis, it has been observed that EVA predicts the risk of cardiometabolic diseases and hypertension. Previous findings support the relationship between CVD and metabolic diseases with the vascular aging process, highlighting the importance of using multiple parameters for accurate assessment [27,28]. Changes associated with the EVA affect both vascular structure and function. Structurally, an increase in the thickness and a reduction in the diameter of the arteries have been reported. Functionally, EVA compromises endothelium-mediated vasodilation capacity and increases arterial stiffness [27]. Furthermore, there is a clear association between EVA and hypertension, reinforcing its role as a relevant marker in the prediction of adverse cardiovascular events [29]. In this context, EVA it is argued to be an independent predictor of both cerebrovascular disease, cardiovascular death and death from other causes [28]. Notably, this evidence is consistent with the concept of a bidirectional relationship between arterial stiffness and hypertension, where each condition contributes to the progression of the other, as previously proposed in the literature [21].

Similarly, a significant trend is observed with a-PWV, which is associated with both cardiometabolic diseases and hypertension, according to our analysis, and is a good predictor of risk. A diagnosis of arterial stiffness assessed by a-PWV is also a strong predictor of CVD risk [30,31]. Arterial stiffness is related to the loss of arterial elasticity and endothelial dysfunction [32]. Recent evidence further supports the clinical relevance of vascular aging assessment in cardiovascular prevention. Vascular aging reflects the cumulative effects of cardiovascular risk factors on the arterial wall and emphasizes the importance of integrating measures such as a-PWV and other vascular biomarkers into routine clinical practice for early risk stratification [33]. Likewise, vascular aging markers can be used to identify early vascular alterations in young adults, even before the development of overt hypertension, suggesting that vascular aging assessment may provide additional information beyond conventional blood pressure criteria [34]. These findings are consistent with our results, which show that EVA and a-PWV are associated with an increased risk of cardiometabolic diseases and hypertension, reinforcing their value as early indicators of vascular damage and cardiovascular risk. On the other hand, according to our analyses, PP is considered a predictor of hypertension risk, reinforcing its usefulness as a key measure in cardiovascular risk assessment. Studies use the SBP to assess the risk of hypertension, which can lead to arterial wall stiffness and thickening. Therefore, PP could be useful in this context [19].

In terms of HbA1c, no statistically significant differences were observed in our analysis. Instead, the evidence supports that HbA1c is a risk factor for mortality and CVD, although previous evidence has reported that an increase above 6% in HbA1c in the nondiabetic population is an indicator of cardiovascular mortality [16]. On the other hand, regarding AGEs, no association was observed with the prediction of the risk of cardiometabolic diseases and hypertension. However, studies estimate that AGEs may be a strong predictor of cardiovascular mortality. Pathophysiological mechanisms include vascular wall stiffening, vascular distensibility and even multiple vascular and myocardial changes [35]. In this analysis, the lack of association of both HbA1c and AGEs could be explained by the sample size, as the number of subjects was not large.

On the basis of the data obtained in this study, EVA and a-PWV are associated with cardiometabolic diseases and hypertension in females; similarly, PP is associated with hypertension in females. Several studies suggest that females may be more susceptible to damage caused by high BP, highlighting the importance of assessing vascular aging from a gender perspective [29,36]. Evidence suggests that as females age, they are more at risk of developing hypertension, particularly at the onset of menopause [37]. Female sex hormones, especially estrogens, have been shown to have a cardioprotective effect [38].

In the adjusted models of this analysis, as more covariates were incorporated, some associations lost statistical significance, as seen with EVA in the total population studied. Age is a key determinant of vascular aging, with increased risks of conditions such as ischemic heart disease, stroke, CVD and left ventricular hypertrophy. Additionally, obesity accelerates vascular aging by increasing arterial stiffness [19,28]. Individuals with lower levels of education often have a lower socioeconomic profile and engage in behaviors associated with poor vascular health, including unhealthy diets, obesity, smoking, and sedentary lifestyles. Similarly, a family history of stroke is linked to a higher risk of cerebrovascular events [39,40]. Finally, smoking is another major contributor to accelerated vascular aging, as it significantly increases the risk of atherosclerosis and other cardiovascular complications. However, smoking cessation has been demonstrated to markedly reduce the risk of vascular disease [28]. The loss of statistical significance in the adjusted models may be explained by the strong correlation between these variables and the outcome of interest, suggesting that they play a crucial role in development.

### 4.1. Future Perspectives and Clinical Implications

The findings of this study have important clinical implications for the early detection and prevention of cardiometabolic diseases and hypertension. The demonstrated association between EVA and its related parameters (a-PWV and PP) with the onset of these events at the 2-year follow-up highlights the potential of incorporating vascular aging assessment into routine clinical practice for early risk detection. In particular, the use of simple, noninvasive, and widely available markers, such as a-PWV and pulse pressure, could allow for the identification of individuals at higher risk even before the development of the disease. Furthermore, the stronger associations observed in women suggest that sex-specific strategies should be considered in cardiovascular risk and vascular health assessments. Together, these results support the integration of EVA-related parameters into cardiovascular risk stratification models, which could contribute to more personalized and timely interventions aimed at reducing the burden of cardiometabolic diseases and hypertension.

### 4.2. Limitations

This study has several limitations that should be considered. First, the EVasCu study adopted a longitudinal design, with a 2-year follow-up cohort, during which 190 participants were lost to follow-up. The study was conducted in Cuenca (Spain), a city characterized by high population mobility due to migration related to education and employment, especially among young people and working-age adults, which may partly explain the loss of participants. Second, although the sample size was acceptable, a larger sample size could have yielded more statistically significant associations. Additionally, the EVasCu study focused on a specific Spanish population, which limits its generalizability to other populations, unless the sociodemographic characteristics are similar to those of the study population. Although the study provides strong evidence that EVA, a-PWV and PP are related to cardiometabolic diseases and hypertension, these considerations should be considered when interpreting the results, and more research is needed in this field, including long-term studies and in different types of populations with large sample sizes.

## 5. Conclusions

On the basis of the results obtained from the analysis of the EVasCu study data, it can be concluded that EVA is associated with the development of cardiometabolic diseases and hypertension at 2-years, especially in females. The parameters included in the EVA construct, specifically a-PWV and PP, are also associated with these diseases, being predominant in females. These findings are of great clinical relevance, since in view of the results obtained, there is ample evidence to identify and carry out risk assessment and preventive strategies. Furthermore, treatment of these diseases and prevention of the relevant risk factors are effective in preventing vascular aging. However, further research is needed to better understand the mechanisms underlying this association, through more years of population follow-up and a larger sample size, as well as working with different populations.

## Figures and Tables

**Table 1:** Comparison of clinical characteristics between baseline and 2-year follow-up in the EVasCu study.

	Baseline				2-Year Follow-Up			
	All Population (*n* = 390)	Male (*n* = 144)	Female (*n* = 246)	*p* Value	All Population (*n* = 200)	Male (*n* = 74)	Female (*n* = 126)	*p* Value
Age (years)	42.02 ± 13.14	42.33 ± 12.45	41.84 ± 13.55	0.722	46.45 ± 12.37	47.96 ± 11.97	45.56 ± 12.56	0.196
Body fat (%)	27.25 ± 9.36	19.92 ± 6.91	31.54 ± 7.82	<0.001 *	-	-	-	-
Family history, *n* (%) Stroke Acute myocardial infarction	65 (17.2) 102 (26.7)	15 (10.6) 45 (31.9)	50 (21) 57 (23.7)	0.010 * 0.078	43 (21.5) 49 (24.5)	10 (13.5) 23 (31.1)	33 (26.2) 26 (20.6)	0.041 * 0.152
Current smokers, *n* (%)	49 (12.6)	15 (10.4)	34 (13.8)	0.627	20 (10)	5 (6.8)	15 (11.9)	0.260
Educational level, *n* (%) Cannot read or write/No studies Primary education Secondary education/Professional formation/High school University degree	0 (0) 49 (12.7) 116 (29.7) 221 (57.3)	0 (0) 20 (14.0) 40 (27.8) 83 (57.6)	0 (0) 29 (11.9) 76 (30.9) 138 (56.8)	0.773	0 (0) 12 (6) 46 (23.0) 141 (99.5)	0 (0) 7 (9.5) 16 (21.6) 51 (68.9)	0 (0) 5 (4) 30 (23.8) 90 (71.4)	0.275
Heart rate (bpm)	67.13 ± 10.82	63.35 ± 10.17	69.31 ± 10.60	<0.001 *	69.38 ± 11.68	65.88 ± 10.82	71.42 ± 11.72	0.001 *
Systolic blood pressure (mmHg)	116.67 ± 15.21	125.09 ± 12.76	111.77 ± 14.37	<0.001 *	118.59 ± 13.78	127.07 ± 12.50	113.64 ± 12.00	<0.001 *
Diastolic blood pressure (mmHg)	70.36 ± 10.57	72.27 ± 10.32	69.24 ± 10.58	0.006 *	71.65 ± 8.91	73.84 ± 8.71	70.38 ± 8.81	0.014 *
Aortic pulse wave velocity (m/s)	6.34 ± 1.35	6.53 ± 1.34	6.23 ± 1.35	0.037 *	6.75 ± 1.45	7.16 ± 1.49	6.51 ± 1.38	0.003 *
Advanced glycation end products (Au)	1.89 + 0.41	1.90 ± 0.46	1.87 ± 0.38	0.489	1.85 ± 0.42	1.85 ± 0.49	1.85 ± 0.37	0.921
Pulse pressure (mmHg)	46.19 ± 10.19	52.45 ± 9.76	42.50 ± 8.50	<0.001 *	46.93 ± 10.05	53.22 ± 10.66	43.26 ± 7.60	0.011 *
Glycated hemoglobin A1c (mg/dL)	5.18 ± 0.33	5.16 ± 0.37	5.19 ± 0.30	0.376	5.24 ± 0.37	5.21 ± 0.35	5.26 ± 0.39	0.582
Construct, *n* (%) Early vascular aging Healthy vascular aging	164 (42.1) 226 (57.9)	65 (45.1) 79 (54.9)	99 (40.2) 147 (59.8)	0.345	89 (44.5) 111 (55.5)	39 (52.7) 35 (47.3)	50 (39.7) 76 (60.3)	0.054
*Cardiometabolic disease patients*	0	0	0	-	25 (12.5)	10 (13.5)	15 (11.9)	0.698
*Hypertensive patients*	0	0	0	-	7 (3.5)	4 (5.4)	3 (2.4)	0.248

Data are presented as mean ± standard deviation (SD) or number of subjects (percentage). * *p* < 0.05 was considered statistically significant.

**Table 2:** Associations between early vascular aging and cardiometabolic diseases and hypertension.

	β ± SE	OR (CIs)	*p* Value	β ± SE	OR (CIs)	*p* Value	β ± SE	OR (CIs)	*p* Value	β ± SE	OR (CIs)	*p* Value
All population												
Cardiometabolic diseases	0.323 ± 0.147	1.382 (1.035–1.844)	0.028 *	0.165 ± 0.192	1.179 (0.810–1.717)	0.391	0.122 ± 0.200	1.130 (0.764–1.672)	0.541	0.211 ± 0.212	1.235 (0.814–1.872)	0.321
Hypertension	0.494 ± 0.203	1.639 (1.101–2.439)	0.015 *	0.375 ± 0.285	1.455 (0.833–2.543)	0.188	0.366 ± 0.301	1.442 (0.800–2.599)	0.223	0.629 ± 0.357	1.876 (0.931–3.779)	0.078
Male												
Cardiometabolic diseases	0.129 ± 0.206	1.138 (0.760–1.704)	0.532	0.034 ± 0.265	1.035 (0.615–1.741)	0.897	−0.067 ± 0.283	0.935 (0.537–1.630)	0.813	0.072 ± 0.307	1.074 (0.589–1.960)	0.815
Hypertension	0.099 ± 0.310	1.104 (0.602–2.026)	0.749	0.040 ± 0.396	1.041 (0.479–2.264)	0.919	−0.063 ± 0.449	0.939 (0.390–2.263)	0.889	0.303 ± 0.608	1.354 (0.411–4.460)	0.618
Female												
Cardiometabolic diseases	0.650 ± 0.251	1.915 (1.171–3.132)	0.010 *	0.446 ± 0.307	1.562 (0.855–2.852)	0.147	0.445 ± 0.312	1.560 (0.846–2.876)	0.154	0.470 ± 0.326	1.599 (0.843–3.032)	0.150
Hypertension	1.405 ± 0.478	4.075 (1.598–10.394)	0.003 *	1.329 ± 0.611	3.777 (1.140–12.515)	0.030 *	1.211 ± 0.620	3.357 (0.997–11.308)	0.051	3.231 ± 2.154	25.308 (0.372–1723.742)	0.134

β (regression coefficient), SE (standard error), OR (odds ratio), and CI (confidence interval). * *p* < 0.05 was considered statistically significant. Model 1: Adjusted for age and gender. Model 2: Model 1 plus body fat percentage and educational level. Model 3: Model 1, Model 2 plus family history and smoking.

**Table 3:** Associations between aortic pulse wave velocity and cardiometabolic diseases and hypertension.

	Unadjusted			Model 1			Model 2			Model 3		
	β ± SE	OR (CIs)	*p* Value	β ± SE	OR (CIs)	*p* Value	β ± SE	OR (CIs)	*p* Value	β ± SE	OR (CIs)	*p* Value
All population												
Cardiometabolic diseases	0.426 ± 0.146	1.531 (1.149–2.038)	0.004 *	0.605 ± 0.337	1.832 (0.946–3.546)	0.072	0.577 ± 0.342	1.781 (0.919–3.485)	0.092	0.696 ± 0.365	2.006 (0.981–4.103)	0.056
Hypertension	0.736 ± 0.234	2.088 (1.319–3.304)	0.002 *	1.716 ± 0.565	5.564 (1.838–16.847)	0.002 *	1.735 ± 0.584	5.668 (1.805–17.797)	0.003 *	2.976 ± 0.897	19.614 (3.380–113.821)	<0.001 *
Male												
Cardiometabolic diseases	0.241 ± 0.221	1.273 (0.825–1.963)	0.275	0.493 ± 0.594	1.637 (0.511–5.244)	0.407	0.309 ± 0.628	1.362 (0.398–4.663)	0.623	0.675 ± 0.702	1.965 (0.497–7.773)	0.336
Hypertension	0.304 ± 0.314	1.356 (0.733–2.507)	0.332	1.228 ± 0.781	3.416 (0.739–15.790)	0.116	1.304 ± 0.837	3.685 (0.714–19.919)	0.119	4.224 ± 2.386	68.320 (0.636–7340.234)	0.077
Female												
Cardiometabolic diseases	0.601 ± 0.217	1.824 (1.192–2.790)	0.006 *	0.737 ± 0.442	2.089 (0.878–4.973)	0.096	0.746 ± 0.451	2.109 (0.871–5.106)	0.098	0.742 ± 0.467	2.101 (0.842–5.243)	0.112
Hypertension	1.495 ± 0.579	4.458 (1.434–13.863)	0.010 *	2.470 ± 1.206	11.817 (1.111–125.642)	0.041 *	2.275 ± 1.210	9.729 (0.908–104.222)	0.060	2.668 ± 1.235	14.411 (1.282–162.021)	0.031 *

β (regression coefficient), SE (standard error), OR (odds ratio), and CI (confidence interval). * *p* < 0.05 was considered statistically significant. Model 1: Adjusted for age and gender. Model 2: Model 1 plus body fat percentage and educational level. Model 3: Model 1, Model 2 plus family history and smoking.

**Table 4:** Comparison of EVA Construct Characteristics at Baseline and 2-Year Follow-Up. Including Absolute Changes (Δ).

	*Baseline*			*2-Year Follow-Up*			Δ					
	All Population (*n* = 390)	Male (*n* = 144)	Female (*n* = 246)	All Population (*n* = 200)	Male (*n* = 74)	Female (*n* = 126)	All Population (*n* = 200)	*p*-Value	Male (*n* = 74)	*p*-Value	Female (*n* = 126)	*p*-Value
Aortic pulse wave velocity (m/s)	6.34 ± 1.35	6.53 ± 1.34	6.23 ± 1.35	6.75 ± 1.45	7.16 ± 1.49	6.51 ± 1.38	0.18	<0.001 *	0.24	<0.001 *	0.15	<0.001 *
Advanced glycation end products (Au)	1.89 + 0.41	1.90 ± 0.46	1.87 ± 0.38	1.85 ± 0.42	1.85 ± 0.49	1.85 ± 0.37	−0.05	0.082	−0.12	<0.001 *	−0.01	0.451
Pulse pressure (mmHg)	46.19 ± 10.19	52.45 ± 9.76	42.50 ± 8.50	46.93 ± 10.05	53.22 ± 10.66	43.26 ± 7.60	0.14	0.851	0.45	0.643	1.27	0.063
Glycated hemoglobin A1c (mg/dL)	5.18 ± 0.33	5.16 ± 0.37	5.19 ± 0.30	5.24 ± 0.37	5.21 ± 0.35	5.26 ± 0.39	0.03	0.082	0.03	0.228	0.04	0.134

Data are presented as mean ± standard deviation (SD). Δ: Absolute Changes—Delta (end-basal). * *p* < 0.05 was considered statistically significant.

**Table 5:** Comparison of cardiovascular parameters between EVA and HVA at baseline and 2-year follow-up. Including Absolute Changes (Δ).

	*Baseline*			*2-Year Follow-Up*			Δ	*p-Value*
	EVA (*n* = 164)	HVA (*n* = 226)	*p* Value	EVA (*n* = 89)	HVA (*n* = 111)	*p* Value		
Aortic pulse wave velocity (m/s)	7.41 ± 1.15	5.56 ± 0.86	<0.001 *	7.85 ± 1.22	5.89 ± 0.95	<0.001 *	−0.06	0.323
Advanced glycation end products (Au)	2.20 ± 0.37	1.65 ± 0.25	<0.001 *	2.10 ± 0.45	0.68 ± 0.54	<0.001 *	0.16	0.665
Pulse pressure (mmHg)	48.94 ± 10.80	44.20 ± 9.23	<0.001 *	49.61 ± 10.63	44.79 ± 9.06	<0.001 *	1.01	0.369
Glycated hemoglobin A1c (mg/dL)	5.39 ± 0.29	5.02 ± 0.25	<0.001 *	5.39 ± 0.29	5.11 ± 0.39	<0.001 *	0.04	0.327
Heart rate (bpm)	66.58 ± 9.38	67.52 ± 11.76	0.200	69.01 ± 10.19	69.67 ± 12.79	0.696	−1.37	0.304
Systolic blood pressure (mmHg)	122.61 ± 16.64	112.32 ± 12.47	<0.001 *	124.16 ± 14.84	114.13 ± 11.07	<0.001 *	0.46	0.765
Diastolic blood pressure (mmHg)	73.33 ± 11.11	68.21 ± 9.64	<0.001 *	74.55 ± 9.08	69.34 ± 8.10	<0.001 *	−0.55	0.628

Data are presented as mean ± standard deviation (SD). Early vascular aging (EVA), Healthy vascular ageing (HVA). Δ: Absolute Changes—Delta ((HVA end–HVA basal)–(EVA end–EVA basal)). * *p* < 0.05 was considered statistically significant.

## Data Availability

The original contributions presented in the study are included in the article; further inquiries can be directed to the corresponding author.

## Supplementary Materials

The following supporting information can be downloaded at: https://www.mdpi.com/article/10.3390/jcm15145520/s1. Table S1. Associations between pulse pressure and cardiometabolic diseases and hypertension; Table S2. Associations between advanced glycation end products and cardiometabolic diseases and hypertension; Table S3. Associations between glycated hemoglobin A1c and cardiometabolic diseases and hypertension.
