# Supplementary material for: Association Between Early Vascular Aging and Cardiometabolic Diseases: A Two-Year Longitudinal Study of the EVasCu Cohort
Source: J Clin Med. 2026 Jul 14;15(14):5520. doi: 10.3390/jcm15145520 (PMC13412954; doi:10.3390/jcm15145520)
Supplement: Supplementary file 1 [file jcm-15-05520-s001.zip › jcm-4364646-supplementary.pdf]

Table S1. Associations between pulse pressure and cardiometabolic diseases and hypertension.

	**Unadjusted**			**Model 1**			**Model 2**			**Model 3**		
	**β±SE**	**OR (CIs)**	**p value**	**β±SE**	**OR (CIs)**	**p value**	**β±SE**	**OR (CIs)**	**p value**	**β±SE**	**OR (CIs)**	**p value**
**All population**												
**Cardiometabolic diseases**	0.018±0.019	1.018 (0.982-1.057)	0.330	0.011±0.021	1.011 (0.969-1.054)	0.620	0.007±0.022	1.007 (0.965-1.051)	0.740	0.011±0.023	1.011 (0.967-1.057)	0.630
**Hypertension**	0.072±0.028	1.074 (1.018-1.134)	0.009*	0.062±0.031	1.064 (1.001-1.131)	0.046*	0.062±0.032	1.064 (1.000-1.134)	0.051	0.086±0.037	1.090 (1.014-1.172)	0.019*
**Male**												
**Cardiometabolic diseases**	0.025±0.030	1.025 (0.966-1.088)	0.413	0.022±0.031	1.023 (0.963-1.086)	0.468	0.014±0.032	1.014 (0.951-1.080)	0.673	0.029±0.036	1.029 (0.959-1.104)	0.428
**Hypertension**	0.033±0.040	1.033 (0.955-1.117)	0.414	0.030±0.040	1.031 (0.953-1.115)	0.448	0.021±0.043	1.021 (0.939-1.110)	0.628	0.043±0.054	1.044 (0.940-1.160)	0.422
**Female**												
**Cardiometabolic diseases**	0.015±0.029	1.015 (0.959-1.075)	0.610	-0.001±0.029	0.999 (0.944-1.058)	0.983	-0.002±0.030	0.998 (0.942-1.058)	0.943	-0.006±0.030	0.994 (0.937-1.055)	0.850
**Hypertension**	0.135±0.051	1.144(1.036-1.264)	0.008*	0.100±0.053	1.105(0.995-1.227)	0.061	0.083±0.057	1.087 (0.972-1.215)	0.142	0.108±0.069	1.114 (0.973-1.275)	0.118

β (regression coefficient), SE (standard error), OR (odds ratio), and CI (confidence interval). * $p<0.05$ was considered statistically significant

Model 1: Adjusted for age and gender. Model 2: Model 1 plus body fat percentage and educational level. Model 3: Model 1, Model 2 plus family history and smoking.

Table S2. Associations between advanced glycation end products and cardiometabolic diseases and hypertension.

	Unadjusted			Model 1			Model 2			Model 3		
	**β±SE**	**OR (CIs)**	**p value**	**β±SE**	**OR (CIs)**	**p value**	**β±SE**	**OR (CIs)**	**p value**	**β±SE**	**OR (CIs)**	**p value**
**All population**												
**Cardiometabolic diseases**	0.441±0.462	1.554 (0.628-3.843)	0.340	-0.224±0.596	0.799 (0.249-2.570)	0.707	-0.374±0.617	0.688 (0.205-2.306)	0.544	-0.373±0.601	0.689 (0.212-2.239)	0.535
**Hypertension**	0.332±0.822	1.394 (0.278-6.987)	0.686	-0.902±1.195	0.406 (0.039-4.216)	0.450	-1.110±1.236	0.330 (0.029-3.718)	0.369	-0.931±1.144	0.394 (0.042-3.708)	0.416
**Male**												
**Cardiometabolic diseases**	0.414±0.619	1.512 (0.449-5.089)	0.504	0.182±0.815	1.200 (0.243-5.932)	0.823	-0.076±0.850	0.926 (0.175-4.906)	0.928	0.048±0.850	1.049 (0.198-5.553)	0.955
**Hypertension**	-0.009±1.043	0.991 (0.128-7.653)	0.993	-0.390±1.416	0.677 (0.042-10.858)	0.783	-0.624±1.373	0.536 (0.036-7.904)	0.650	-1.729±2.177	0.177 (0.002- 12.643)	0.427
**Female**												
**Cardiometabolic diseases**	0.443±0.717	1.557 (0.382-6.352)	0.537	-0.441±0.902	0.643 (0.110-3.770)	0.625	-0.494±0.927	0.610 (0.099-3.752)	0.594	-0.628±0.944	0.534 (0.084-3.395)	0.506
**Hypertension**	0.628±1.426	1.874 (0.115-30.661)	0.660	-1.425±2.381	0.241 (0.002-25.570)	0.549	-1.207±1.966	0.299 (0.006-14.110)	0.539	-1.178±2.277	0.308 (0.004-26.697)	0.605

β (regression coefficient), SE (standard error), OR (odds ratio), and CI (confidence interval). * $p<0.05$ was considered statistically significant

Model 1: Adjusted for age and gender. Model 2: Model 1 plus body fat percentage and educational level. Model 3: Model 1, Model 2 plus family history and smoking.

Table S3. Associations between glycated hemoglobin A1c and cardiometabolic diseases and hypertension.

	Unadjusted			Model 1			Model 2			Model 3		
	**β±SE**	**OR (CIs)**	**p value**	**β±SE**	**OR (CIs)**	**p value**	**β±SE**	**OR (CIs)**	**p value**	**β±SE**	**OR (CIs)**	**p value**
**All population**												
**Cardiometabolic diseases**	0.818±0.632	2.266 (0.657-7.816)	0.196	0.265±0.693	1.304 (0.335-5.073)	0.702	0.183±0.696	1.201 (0.307-4.697)	0.793	0.422±0.738	1.526 (0.359-6.482)	0.567
**Hypertension**	0.302±1.169	1.353 (0.137-13.382)	0.796	-0.467±1.248	0.627 (0.054-7.234)	0.708	-0.514±1.251	0.598 (0.051-6.951)	0.681	-0.219±1.410	0.803 (0.051-12.736)	0.877
**Male**												
**Cardiometabolic diseases**	0.743±0.830	2.102 (0.413-10.702)	0.371	0.547±0.919	1.727 (0.285-10.466)	0.552	0.570±0.916	1.769 (0.293-10.661)	0.534	0.885±1.003	2.423 (0.340-17.288)	0.377
**Hypertension**	-1.241±1.587	0.289 (0.013-6.478)	0.434	-1.910±1.905	0.148 (0.004-6.200)	0.316	-1.974±2.012	0.139 (0.003-7.167)	0.327	-5.047±3.274	0.006 (0.000-3.936)	0.123
**Female**												
**Cardiometabolic diseases**	0.920±0.965	2.509 (0.379-16.617)	0.340	0.038±1.039	1.039 (0.136-7.955)	0.971	-0.009±1.077	0.991 (0.120-8.180)	0.993	0.125±1.117	1.133 (0.127-10.120)	0.911
**Hypertension**	3.342±2.073	28.282 (0.487-1643.130)	0.107)	1.830±2.268	6.235 (0.073-531.118)	0.420	3.224±2.562	25.139 (0.166-3810.571)	0.208	7.958±4.780	2856.996 (0.244-33479780.947)	0.096

β (regression coefficient), SE (standard error), OR (odds ratio), and CI (confidence interval). * $p<0.05$ was considered statistically significant

Model 1: Adjusted for age and gender. Model 2: Model 1 plus body fat percentage and educational level. Model 3: Model 1, Model 2 plus family history and smoking.
